# Molecular Interactions of β-(1→3)-Glucans with Their Receptors

**DOI:** 10.3390/molecules20069745

**Published:** 2015-05-27

**Authors:** Laurent Legentil, Franck Paris, Caroline Ballet, Sophie Trouvelot, Xavier Daire, Vaclav Vetvicka, Vincent Ferrières

**Affiliations:** 1 Ecole Nationale Supérieure de Chimie de Rennes, CNRS, UMR 6226, 11 Allée de Beaulieu, CS 50837, 35708 Rennes Cedex 7, France; E-Mails: laurent.legentil@ensc-rennes.fr (L.L.); franck.paris@ensc-rennes.fr (F.P.); caroline.ballet@ensc-rennes.fr (C.B.); 2 Université européenne de Bretagne, F-35000 Rennes, France; 3 INRA, UMR AgroSup/INRA/uB 1347 Agroécologie, Pôle Interactions Plantes-Microorganismes-ERL CNRS 6300, 21065 Dijon Cedex, France; E-Mails: sophie.trouvelot@u-bourgogne.fr (S.T.); xavier.daire@dijon-inra.fr (X.D.); 4 Department of Pathology, University of Louisville, Louisville, KY 40202, USA

**Keywords:** β-(1→3)-glucans, Dectin-1, CR3, glycolipids, langerin, CBM

## Abstract

β-(1→3)-Glucans can be found as structural polysaccharides in cereals, in algae or as exo-polysaccharides secreted on the surfaces of mushrooms or fungi. Research has now established that β-(1→3)-glucans can trigger different immune responses and act as efficient immunostimulating agents. They constitute prevalent sources of carbons for microorganisms after subsequent recognition by digesting enzymes. Nevertheless, mechanisms associated with both roles are not yet clearly understood. This review focuses on the variety of elucidated molecular interactions that involve these natural or synthetic polysaccharides and their receptors, *i.e.*, Dectin-1, CR3, glycolipids, langerin and carbohydrate-binding modules.

## 1. Introduction

β-(1→3)-Glucans are homopolymers of glucose where each unit is linked to the next by a β-(1→3) bond. They are known to interact with various receptors in humans, invertebrates or microorganisms. Such recognition triggered various responses like immune cascade or digestion. Strangely, most receptors that specifically recognize β-(1→3)-glucans share common characteristics and some show great homology. The mechanisms involved in immunological and inflammatory responses induced by β-glucans or the degradation of β-glucans are complex. They may depend on several factors: characteristics of the β-(1→3)-glucan (molecular weight, degree of branching, solubility, 3D-conformation) and how glucans interact with their receptors. Amongst the latter, the main receptors are Dectin-1, complement receptor 3 (CR3), glycolipids or Carbohydrate Binding Module (CBM) associated with glucanases. Many reviews very interestingly describe a macroscopic view of the resulting biological effects [1,2]. Since information was widely accumulated during the past decade for humans or invertebrates [3], we propose herein an overview of the molecular interactions between these natural polysaccharides or synthetic analogs and their receptors, or mutants thereof.

## 2. Conformation of β-(1→3)-Glucans

The structural conformations of β-(1→3)-glucans in solution cannot be known precisely. This is mainly due to free and independent rotation of glycosidic bonds between residues and the interconversion of these residues between chair, half chair and boat cyclic conformations. As a result, these polysaccharides can be seen as random coils in solution. Computer assisted model building was used to predict the conformations of highest probability that possess a minimum of conformational energy, and where the coils are stabilized by intermolecular interactions. β-(1→3)-glucans were shown to exhibit preferentially a right or left-handed triple helix configuration in water. This was confirmed by experimental studies such as X-ray fiber diffraction [4], solid state ^13^C-NMR spectroscopy [5], multi-angle laser light scattering [6], fluorescence resonance energy transfer spectroscopy [7] and molecular dynamic simulation [8]. In the triple helical structure of β-(1→3)-glucans, the C-2 hydroxyl group of each glucose unit plays an important role in stabilizing the structure by interstrand hydrogen bonding. On the other hand, the primary hydroxyl is oriented toward the outside of the helical structure without any intrastructural interaction. This is why chemical modifications of β-(1→3)-glucans preferentially occur on the branched glucosyl residues or the 6 position: OH-2 and OH-4 connected to the main-chain are required to stabilize the inherent helical structure [9]. Interestingly, it has been established that the chemical modification of the C-6 position scarcely reduces the helix-forming character of β-(1→3)-glucan polysaccharides [10]. To provide an order of magnitude, X-ray diffraction patterns of curdlan [strictly linear β-(1→3)-glucan] in the anhydrous form revealed that it adopts a right-handed 6_1_ triple helix with a diameter of 2.3 nm and a pitch of 1.84 nm. This corresponds to six glucose residues in one helix turn—every 1.84 nm [9].

When dissolved in an alkaline medium (pH higher than 13), in DMSO or at temperature higher than 135 °C, those cooperative interactions are lost. The polysaccharidic chains then adopt less organized conformations and form random coils. The chains can be reconstructed by mixing the solution with excess water [10]. The random coil can therefore be converted into a single helix by neutralization with HCl. Finally, the single helical conformation can be renatured to the original triple helix by heating or dialysis [11]. When renatured, some β-(1→3)-glucans, such as scleroglucan, are able to adopt unusual conformations, for instance circular triple helices structures [12]. The possible mechanism is that closed triple helices are converted into single strand by partially opening one end of the triple helix with sodium hydroxide. Subsequent neutralization returns it to the original closed triple helix. The partially opened structure can be maintained for up to 20 days [7,13]. The significant differences between the triple- and single-helix or partially opened conformations include the accessibility to OH-2 functions of the main-chain strand for further chemical modification [14].

The degree of polymerization (DP) of β-(1→3)-glucans is a determinant factor in their solubility in water. Unbranched laminarins with DPs lower than 20 are freely soluble in water [15]. However, linear β-(1→3)-glucosides derived from curdlan with DPs above 36 are insoluble in water because the cooperative interactions between chains becomes stronger than those between chains and water molecules [16]. However, when these polysaccharides bear (1→6)-linked glucosyl chains, like scleroglucan and schyzophyllan (DP above 100), their water solubility increases. This solubility depends on the frequency of side branches [17]. Removing those residues causes the polysaccharide to precipitate [18]. Finally, high molecular weight (DP > 1000) β-(1→3), (1→6)-glucans from yeast and fungal cell walls are insoluble in water. Solubility of β-(1→3)-glucans is better in alkaline media than in neutral water. This is due to the ionization of hydroxyl groups, disrupting the ordered structures. Organic solvents with the ability to form hydrogen bonds with the polysaccharidic chains such as DMSO and DMF are also good solvents for β-(1→3)-glucans.

It is essential to note that in biological systems, the gel state is the most typical state for β-(1→3)-glucans, ranging from compacted polysaccharides with low hydration, such as callose deposit, to expanded gels like the cell wall matrices of higher plants.

## 3. Interactions of β-(1→3)-Glucans with Human Main Receptors

### 3.1. Dectin-1

Dectin-1 [19] is a type II transmembrane protein present in leukocytes, with the highest levels of cell surface expression in neutrophils, macrophages, and dendritic cells. The human protein consists of 247 amino acids and three major domains [20]. By interacting with a particulate glucan, an immunoreceptor tyrosine-based activating motif (ITAM) is phosphorylated [21]. This transmembrane signaling triggers several biological effects, including the production of superoxide, increase of phagocytosis, and induction of cytokines or anti-fungal effectors [22]. Recently, it was also established that intraocular administration of particulate β-(1→3)-glucans induced axonal regeneration through interaction with Dectin-1 presenting retina-resident immune cells [23]. Interestingly, β-(1→3)-glucans should interact with two surface Dectin-1 receptors to induce intracellular Syk activation, and then formation of phagosomes for cellular internalization and mature phagolysosomes [24]. A Dectin-1/Syk/NF-*k*B signaling axis was further confirmed by experiments using glucan isolated from the fruit bodies of *Grifola frondosa*. The homogeneous β-glucans induce the activation of Syk and NF-*k*B signaling in macrophages resulting in significant anti-cancer activities [25].

Moreover, the strength of the biological response induced by Dectin-1/glucan interaction could also be dependent on the expression level of TLR2, and on cooperative ability of Dectin-1 and TLR2 to interact with β-(1→3)-glucans [21,26]. Following the internalization of the polysaccharide into the macrophage, Chan and coworkers hypothesized that shorter fragments of β-(1,3)-glucans, but still polymeric, are then released to be further taken up by granulocytes thanks to binding by complement receptor-3 (CR3; See corresponding chapter) [27].

Glucan/Dectin-1 interaction is made possible thanks to the presence of a key C-type lectin (CTL) on Dectin-1 as shown by Ohno and coworkers in 2004 [14]. Generally, ligation of CTL with galactosidic or mannosidic ligands requires at least a highly conserved sequence of three consecutive amino-acids within the carbohydrate recognition domain (CRD) of C-type lectins: glutamine-proline-aspartate (QPD) or glutamate-proline-asparagine (EPN), respectively [22]. This interaction is generally calcium-dependent since Ca^2+^ cations are likely to form a complex with both the peptidic sequence and the galactosyl or mannosyl residues [28]. Nevertheless, β-(1→3)-glucans are also recognized by Dectin-1 through a Ca^2+^-independent mode, suggesting modification of the C-type lectin action and so a different peptidic requirement. As nicely shown by Ohno, the glucan binding to the studied receptor involves two amino-acid residues, *i.e.*, a tryptophan (W) and a histidine (H), separated by a third residue. Mutations of one or both residues significantly affected the ability of various glucans to bind Dectin-1.

Parallel to this study, binding mode with glycoconjugates was carried out on collectins, effector molecules of the immune system, which also contain a CTL domain [29]. It was assumed that interactions involving a tripeptide and Ca^2+^ cations are key determinants but that a further pocket was required for larger ligands. Indeed, considering the interactions of Dectin-1 and β-(1→3)-glucans based on a microarray technology, Feizi and coworkers have shown that a minimum of 10 or 11 glucosyl residues were required [30]. This polymer chain length was compatible with a possible defined conformation, so that high order local arrangement was hypothesized. An answer was initially attempted by Ohno who used a simple helix pre-arranged glucan. On a molecular scale, it was concluded that the protein preferred interacting with OH-4 and OH-6, rather than with OH-2. This assumption was later corroborated by STD-NMR technique. Such a NMR sequence is able to give a dynamic view of the contacts between a small molecule and its macromolecular receptor in solution. On this basis, it was established that Dectin-1 induced less contacts with H-2 and H-4 than with H-3, H-5, H-6 and anomeric H-1 [31]. This means that interactions occurred between the hydrophobic face of the glucan and hydrophobic groove of the lectin site through amino acid residues such as histidine and more particularly tryptophan. Nevertheless, it was also shown that both extremities may strongly interact with Dectin-1. Using similar NMR techniques, Yamaguchi and coworkers confirmed that the affinity of oilgo-glucans for Dectin-1 increased with the DP, the stronger binding being measured with laminarin as ligand [32].

Multivalent oligoglucans were recently obtained thanks to controlled grafting of synthetic glucans to proteins like Keyhole limpet hemocyanin (KLH) [33] or detoxified diphtheria toxin (CRM197) [34]. While the laminarihexaose do not bind effectively to Dectin-1, the corresponding multivalent conjugates exhibit strong affinity. In addition, a strong immune response in mice was measured. These results pave the way to the development of β-glucan based vaccine to protect itself against invasive microorganisms like *Candida albicans* or *Aspergillus fumigatus*.

It is well known that the primary structure of β-(1→3)-glucans is significantly dependent on its origin (seaweeds, fungi, yeasts) and isolation protocols. Differences were observed in terms of molecular weight, chain length, and branching. The latter parameter has a strong influence on the conformational behavior of the natural polysaccharides [8]. It was recently demonstrated that the existence of such side-chain (1→6)-branching, and its position, on short oligo-β-(1→3)-glucans may influence binding affinities with Dectin-1, IC_50_ values varying from 2.6 mM to 2.2 pM [35]. Moreover, interaction mechanisms of discontinuous cereal β-(1→3), β-(1→4)-glucans were partly elucidated. It was observed that these polysaccharides have weaker affinities than continuous β-(1→3)-glucans but that helical arrangement was not necessary to ensure interactions with Dectin-1 and that conformational adaptation was possible [36]. In conclusion, no general rules could be clearly drawn to explain elicitation of immunological responses since recognition of Dectin-1, with or without the assistance of TLRs, is strongly linked to the type of β-glucans used [37].

### 3.2. Langerin

Amongst dendritic cells, Langerhans cells are able to present surface langerin that bears a C-type lectin domain. It is, therefore, involved in viruses, fungi and bacteria binding, followed by internalization of the parasites, and induction of immunological responses [38]. Langerin is composed of two main parts: a carbohydrate-recognition domain and a region that can form trimers. Although its ability to complex with mannose-, fucose-, and *N*-acetyl-glucosamine-containing polysaccharide was well known, recent studies established molecular interactions with β-(1→3)-glucans. Geijtenbeek and coworkers showed that langerin is an important receptor for this family of polysaccharides which are commonly found at the surface of opportunistic and pathogenic microorganisms such as *Candida* and *Saccharomyces* species [39]. To complete this study, langerin was co-crystallized with laminaritriose [40]. Crystallographic data highlighted that the non-reducing glucose residue could coordinate calcium cation through hydrogen bonds involving its OH-3 and OH-4 functions, the cation being coordinated within a tweezer consisting of two asparagine and two glutamine residues. The authors also demonstrated the ability of the reducing glucose residue to bind calcium through the free β-anomeric hydroxyl and its vicinal OH-2. They also concluded that such interactions on both reducing and non-reducing extremities induced conformation with minimum energy. Very recently, the transport of glucan particles was studied in the context of gut-associated lymphoid tissue. Once again, CTLs were determined to be suitable receptor, and more particularly that of langerin presented by leukocytes within the intestinal mucosa [41].

### 3.3. CR3

Complement Receptor 3 (CR3) is a receptor belonging to the β_2_-integrin family, found on immune cells such as leukocytes, macrophages and NK cells. As part of the complement system (innate immunity), CR3 is the main receptor involved in clearing iC3b-opsonized fungal pathogens from an organism by stimulating phagocytosis and degranulation. This heterodimeric transmembrane glycoprotein is made up of a common β_2_ subunit (CD18), noncovalently bound to the α_M_ subunit (CD11b) [42]. This α-chain is unique in containing a lectin-like site, involved in priming of CR3 and its proper adhesion conformation [43,44].

β-Glucans can bind with high affinity to the lectin site and the overlapping I-domain of CD11b. However, a double recognition has been shown to be mandatory to trigger the immune system and leading to a cytotoxic activity. This relies on a simultaneous binding of iC3b-opsonized molecules and β-glucan on CR3 [45]. This dual ligation was first highlighted with the use of yeasts that show the polysaccharide within their membrane. Once administered, the fungi pathogen is opsonized by iC3b, and binds the two active sites of CR3, leading to the desired cytotoxic effect [46]. More precisely, β-glucans activate CR3 for binding with iC3b-coated cells, which can be antibody-coated cancer cells, fungi or yeast [27,47]. These findings were confirmed by inhibition by anti-CD11b antibodies and by use of CR3 KO mice [48].

In order to enhance the immune response in humans, unable to synthetize β-(1→3)-glucans, many groups have worked over the past years on the oral [49] or intravenous administration of soluble polysaccharides, synthetic or natural, willing the same cytotoxic effects [50]. Bose’s team showed that soluble β-glucans of 10 kDa could bind human neutrophils and monocytes in a concentration-dependent and receptor-specific manner [51]. It was also proposed, and confirmed later with STD-NMR studies [31], that small soluble oligosaccharides (less than 10 repeating units) cannot bind the lectin site [30]. More recently, Crich and coworkers analyzed the effects of shorter hydroxylamine analogues of β-glucan. Interestingly, they obtained a CR3 activation, hence a phagocytosis activity, suggesting that the number of repeating glucosyl entities is not the only factor modulating the priming of CR3 [52]. The high biological efficiency of these small analogues compared to the activity of longer polysaccharides was assumed to be based on increased hydrophobicity of the α-face of the synthetic 2-deoxy hydroxylamine mimetics. As a matter of fact, it was demonstrated that laminaripentaose, a five-membered oligoglucan, had an immunostimulatory effect *in vivo* in murine models [53]. Other short β-(1→3)-glucan mimetics also triggered immunostimulating potential at least equivalent to that of laminarine, *i.e.*, deoxy-derivatives [54,55,56] or thioglucan analogues [57], the latter being active on cancer stem cells.

### 3.4. Glycolipids, Scavenger Receptors and other Receptors

Lactosylceramide (LacCer, CDw17) is a glycosphingolipid highly expressed on the plasma membranes of human neutrophils and epithelial cells. In 1998, thanks to biochemical interaction studies between PGG-glucan and human leukocytes, Zimmerman and coworkers identified LacCer as a β-(1→3)-glucan receptor [58]. It has been demonstrated thereafter that β-(1→3)-glucan-bounded LacCer triggers various responses in human neutrophil, such as chemotaxis [59], oxidative burst and NF-κB activity [60], and cytokine secretion [61]. All together, these effects lead to the increase of neutrophil antimicrobial activity. However, the mechanisms behind this immune cascade remain unclear [62].

Other lipid-conjugates are also able to interact with β-(1→3)-glucans. Acetylated low density lipoproteins (AcLDLs) presented by human U937 cells however prefer negatively polyglucans [63], for instance phosphorylated ones, or linear glucosyl chains [64]. It was shown that branched schizophyllan had no ligation affinity for LDL. More recently, Lozano and coworkers have highlighted that binding of β-(1→3)-glucan zymosan with the CD5 ectodomain, a receptor present on the surface of mature T and B1a cells and belonging to the scavenger receptor cysteine-rich family, induced MAP kinase activation and cytokine release [65].

Finally, it was recently established that molecular components of inflammasome are able to interact with fungal glucans [66]. The inflammasome is a proteic oligomeric complex involved in the regulation of the innate immunity. It is notably composed of caspases 1 and 5, PYCARD, and proteins of the nucleotide-binding oligomerization domain-like receptors (NLR). Along the latters, the cryopirin, also called NALP3, should be not essential for the production of interleukin-1β but the formation of a complex built of candida β-glucan/Dectin-1/TLR2 proceeded in production of the interleukin. In this context, results obtained by Matikainen and coworkers underlined that b-glucans interacting with cytoplasmic NLRP3 inflammasome triggered IL-1β production in human macrophages [67].

### 3.5. Other Lectins

In addition to major β-(1→3)-glucan receptors Dectin-1 and CR3, other proteins were described as β-(1→3)-glucan recognition protein (βGRP). L-Ficolin is a lectin that participates in the innate immunity cascade *via* the complement activation [68]. It is activated by contact with capsulated bacteria through recognition of a large panel of PAMP (Pathogens Associated Molecular Pattern) including lipoteichoic acid [69], acetylcholine, GlcNAc-containing cell wall [70] and β-(1→3)-glucan [71]. The group of Gaboriaud and coworkers identified a β-(1→3)-glucan binding domain located on two contiguous sub-sites of the trimeric form of the protein [72]. The polymer binds through four glucosyl units between two sides of a cleft formed by sub-sites S3 and S4. The recognition sites adopt a curved conformation that accommodates well with the linear β-(1→3)-glucan. First anchorage of the reducing end occurs at S4 site and is stabilized thanks to hydrophobic interaction between the reducing sugar and a tryptophan. Additional H-bonds are involved, one in particular mediated by a calcium cation. Toward the non-reducing end, direct and water-mediated hydrogen bonds occurred mostly with 2-OH of the glucosyl residue. This pattern is the first described example of contiguous sub-site that recognizes one specific assembly.

## 4. Elicitation of Immune Responses in Invertebrates

### 4.1. Insects Receptors

Insects do not possess acquired immunity. They solely rely on innate immune cascade upon invasion of parasite. Such pathways include prophenoloxidase (proPO) cascade, Toll and immune deficiency pathways [73,74]. They could be triggered when βGBP bind to β-(1→3)-glucan expressed at the surface of invasive parasites [75,76]. Various βGRP have been identified among different species of insect [77,78,79]. They share a common organization with two distinct domains [80]. The *C*-terminal domain is a glucanase-like domain that presents some homology with identified xylanase or starch-hydrolyzing enzyme. It could neither hydrolyze β-(1→3)-glucan nor bind to insoluble glucan [81,82]. In some examples however, some affinity with soluble laminarin was detected [83]. Its absence reduces the immune response drastically but its complete role is still to be determined. The *N*-terminal domain was described as the β-(1→3)-glucan recognition domain. It is made of 100 amino-acids on average and is highly conserved among insect species. Different research groups managed to produce the *N*-terminal domain of βGRP (N-βGBP) from different species as recombinant protein [84,85,86,87,88]. Subsequent NMR and X-ray crystallography analyses highlighted a common structure for all the deciphered N-βGBP. They all have an immunoglobulin like β-sandwich fold composed of two antiparallel β-sheets.

The first model described was the *N*-terminal domain of *Drosophila* Gram-negative binding protein 3 (GNBP3) [88]. GNBP3 is a circulating protein found in the hemolymph of *Drosophila* that senses fungal invasion and subsequently triggers a proteolitic cascade that eventually activates the Toll receptors [89]. Activation of the Toll pathway leads to the production of antimicrobial peptides.

*N*-terminal domain of GNBP3 exhibits strong affinity for linear non-soluble β-(1→3)-glucan curdlan but low binding to oligoglucans with a degree of polymerization (DP) less than 16. Lectin and CBM generally bind weakly to carbohydrates due to low energy-binding bonds involved in the interaction [90,91]. In order to counterpart these non-favorable interactions, two strategies prevailed. In the first case, the receptors agglomerate around the macromolecules thus increasing the affinity by clustering effect. In the second case, the binding surface on the receptors is wide enough to accommodate a large portion of the polysaccharides. Here, Roussel and coworkers [88] identified a large aromatic rich binding platform made of the triad Tyr^75^-Trp^77^-Tyr^79^, which could interact with glucan thanks to hydrophobic interactions. This platform could be accessible to the polysaccharide only if the negatively charged loop, that hides two of the three aromatic amino-acids, is put back. The authors hypothesized that a linear β-(1→3)-glucan with high DP was necessary to open this lid. Once the polysaccharide is in the groove, the loop could close and thus form an additional stabilizing hydrophobic bond with the glucan.

**Figure 1:**
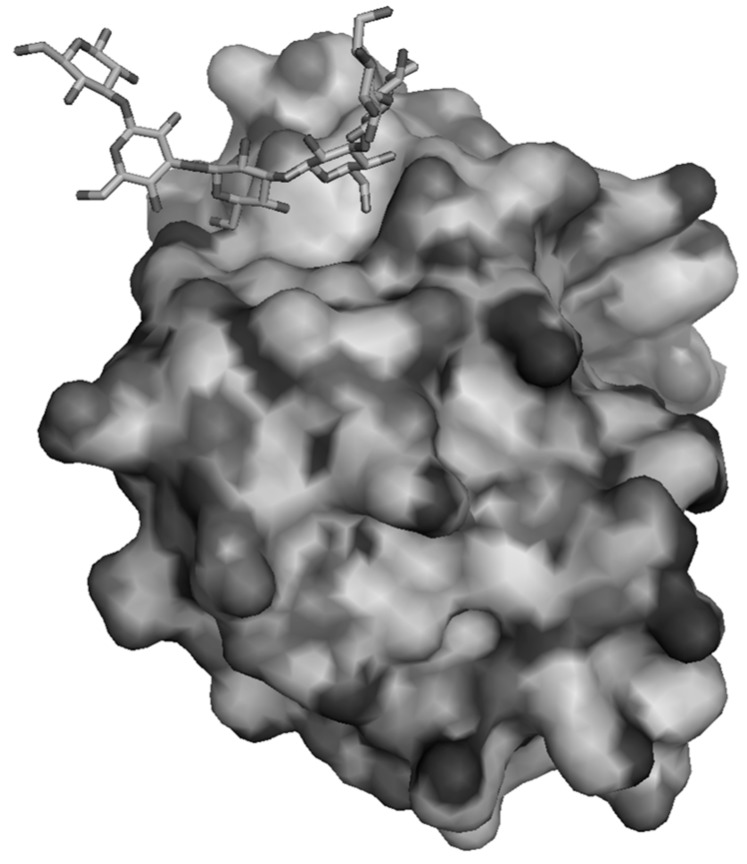
Solvent accessible surface of *Plodia interpunctella* N-βGRP complexed with laminarihexaose (data from Protein Data Bank entry 3AQZ).

Later, Kanagawa and coworkers [86] described the X-ray crystallographic data of the N-βGRP of both *Plodia interpunctella* and *Bombyx mori* in complex with laminarihexaose (Figure 1). Such N-βGRP is able to activate the proPO cascade leading to the encapsulation of the invading micro-organism [83,92]. In the reported data, these authors confirmed the localization of the interaction site on the convex β-sheet of the globular protein like for *Drosophila* and the stabilization of the resulting complex thanks to a long loop between the β3 and β4 strand. These results are in contradiction with previous data on N-βGRP of *B. mori* that localize the site of recognition on the concave *C*-terminal face of the protein by NMR titration in the presence of laminarin [87]. Such discrepancies might be associated with the use of different sources of laminarin. Interestingly, they also highlighted the crucial role of Trp^76^ that interacts with two molecules of laminarihexaose in analogy with Trp^77^ in *Drosophila* [86]. However, the protein appears to have a preformed and rigid site of interaction thus ruling out any adaptation of the receptors at the substrate approach. Three molecules of laminarihexaose interact with each other in the groove to form a β-(1→3)-glucan like macromolecules. β-(1→3)-glucan like curdlan adopts in water a right-handled helical conformation thanks to inter-chain hydrogen bonds between the 2-O groups and intra-strand H-bond between 4-O and 5-O atoms [4]. As such, curdlan or laminaran present a hydrophobic core and a rather hydrophilic surface. There is therefore no need of an aromatic patch that can stack with the carbohydrate residue in the recognition site. Interestingly, X-ray data exhibited two major contributions in the affinity site. First, hydrophobic bonds (and not stacking) are found between 6 glucoses belonging to three strands of laminarohexaose and hydrophobic residue Leu, Trp and Phe. Secondly, polar and charged residues like Hist, Asp and Arg circle the hydrophobic domain and contribute to a stabilizing H-bond network with 4-OH and 6-OH pending at the surface of the “triple helical”-like laminarihexaoses.

The idea of a large site of interaction that can accommodate a large portion of the β-(1→3)-glucan to compensate low-energy binding was recently reinvestigated using ITC and analytical ultracentrifugation techniques [85]. The authors compared the behavior of N-βGRP from *P. interpunctella* in presence of laminarihexaose or laminarin. First, numerous ^1^H-^1^H NOE distance constraints were detected on the non-bound and bound protein, thus validating the conformational rigidity of the protein highlighted previously. The analysis of sedimentation velocity shows the formation of a macro-complex N-βGRP/laminarin where one laminarin bind two proteins. In addition to agglutination of the protein on laminarine, the complex is stabilized thanks to a salt bond between two adjacent proteins. Such complex stability is influenced by the amount of β-(1→6)-branching on the glucan. The authors also hypothesized that the agglutination of βGRP around the molecule of laminarin is a crucial step to trigger the serine protease cascade. Such cluster effect was recently identified as the mechanism involved when soluble laminarin interacted with the *N*-terminal domain of βGRP2 from *Manduca sexta* [84]. At equimolar concentration of the glucan and the protein, an irreversible insoluble aggregate forms that is able to activate the serine protease cascade. Oligomerization of N-βGRP2 around a molecule of laminarin was possible thanks to the packing of proteins in a side-by-side fashion *via* electrostatic interactions similar to the one found for *B. mori* and *P. interpunctella* [85]. This clustering forces the laminarin in solution into a partial triple-helix configuration similar to curdlan and, as such, insoluble in water.

When a larger concentration of the polysaccharide is used, no such precipitate appears, even if a clustering occurs. Dispersion of the protein along the chain of laminarin allows enough flexibility to the polymer to be soluble. Interestingly, this complex shows less activity upon immune cascade activation than the insoluble precipitate. These data give a partial explanation on the observed fact that the immune cascade activation reaches a maximum at a certain concentration of laminarin and then decreases with increasing amounts of sugar [93,94].

### 4.2. Crustacean Receptors

As insects, crustaceans rely on their innate immune system to fight invasive parasites [95,96]. Various pattern recognition proteins are involved in the recognition of epitopes at the surface of the microorganism. They include β-glucan binding or recognition protein (βGBP or βGRP), lipopolysaccharide and β-(1→3)-glucan binding protein (LGBP), Gram-negative binding or recognition protein (GNBP or GNRP) and peptidoglycan binding or recognition protein (PGBP or PGRP) [97,98,99].

The first example of βGBP has been identified in the blood of the shrimp *Pacifastacus leniusculus* [100]. Upon β-(1→3)-glucan binding, it mediates different defense reactions like opsonisation or ProPo cascade. Sequencing shows unique repeating sequences not found in mammalian lectins and a sequence that shows similarity with identified glucanase. Later on, numerous βGBP were purified and cloned in various crustaceans like crayfish [101], shrimp [95] or crab [102]. No systematic analysis of the recognition pattern has yet been published. However, an attempt to understand their specificity for β-(1→3)-glucan using *in silico* docking and molecular dynamics simulation were reported recently. For example, the recognition domain of β-GBP from *Episesarma tetragnonum* was computed by homology with the catalytic domain of a laminarase from *Thermotoga maritima* [103]. The author identified a polar groove at the central core of the protein where only laminarin can fit. Same homology modeling was performed on LGBP isolated from the shrimp *Fenneropenaeus indicus* [104]. Such protein shows no specificity for glucan as it also binds to LPS. It is typically described as a multi-domain protein with sub-sites dedicated to the recognition of specific PAMP and to the cell-adhesion process. This sub-site comprises a binding groove rich in polar and charged amino-acids that seems to fit best with oligo-glucan models.

A last family of β-(1→3)-glucan binding domain were identified as part of the coagulation Factor G of the horseshoe crab [105]. Upon binding with long glucan (DP greater than 7), Factor G dimerized and the resulting activated specie activates the hemolymph clotting cascade. Factor G is a heterodimeric serine protease zymogen comprising two sub-units α and β that are linked through non-covalent bonds [106]. The α-unit presents similar domains as glucanase and it was reported that only the *C*-terminal domain of the unit binds effectively to the glucan [107]. This domain carries two independent glucan-binding sites and certainly interacts with the long chain of β-(1→3)-glucan by multiple interactions. An action mechanism was predicted based on these findings. First detection of fungal β-(1→3)-glucans occurs on both independent sites of the *C*-terminal domain of the α-unit leading to a stable complex. The non-covalent linked β-unit is thus recruited on the surface of the pathogen. Activation into the dimer occurs by intermolecular interaction of two Factor G sliding along the same chain of glucan. Such activation is impossible with short glucans that are not long enough to stabilize the complex and to favor the encounter of the Factor G.

## 5. Recruitment of Glucanases

Glycoside hydrolases play a considerable role in the digestion and recycling of fixed carbon found in polysaccharides (plant cellulose and xylan, algae laminarin, *etc.*). They are generally constituted of a catalytic domain linked to one or more binding domain named Carbohydrate Binding Module (CBM) [108]. Such modules lack catalytic activity but have strong affinity for the target polysaccharide. It allows increasing the concentration of enzyme at the proximity of the glycan and thus positively influencing the rate of their hydrolysis [109,110]. So far, 71 families of CBM have been described based on their amino-acids sequence, binding specificity and ternary structure [111]. They can be further categorized according to the topology of the carbohydrate-binding site [112]. According to CAZY database, β-(1→3)-glucanases could be associated with five different families of CBM (Table 1). Only five modules were successfully crystallized and their structure resolved with or without ligand.

**Table 1:** Isolated CBM associated with β-(1→3)-glucanases. Data available from CAZY database (www.cazy.org) in April 2015 (^a^ X-ray data available).

CBM Family	Catalytic Activity of the Associated Glucanase	Organisms
CBM 4	Endo- β-(1→3)-glucanases	*Paenibacillus* sp.
	(E. C. 3.2.1.39/3.2.1.6)	
	Lichenase A/Laminarinase	*Ruminiclostridium thermocellum* ^a^
	(E.C. 3.2.1.6/3.2.1.73)	*Thermotoga maritime* ^a^
	Endo- β-(1→3)-glucanase	*Thermotoga neapolitana*
	(E. C. 3.2.1.39)	*Thermotoga petrophila*
CBM 6		*Bacillus halodurans* ^a^
	Endo- β-(1→3)-glucanase	*Lysobacter enzymogenes*
	(E. C. 3.2.1.39)	*Streptomyces sioyaensis*
		*Zobellia galactanivorans* ^a^
CBM 43		*Aspergillus fumigatus*
	β-(1→3)-Glucanosyltransglycosylase	*Candida albicans*
	(E.C. 2.4.1.-)	*Pichia pastoris*
		*Paracoccidioides brasiliensis*
		*Saccharomyces cerevisiae* ^a^
		*Schizosaccharomyces pombe*
		*Olea europaea* ^a^
	Endo-β-(1→3)-glucanase	*Pisum sativum*
	(E.C. 3.2.1.39)	*Salix gilgiana*
		*Triticum aestivum*
CBM 52	Endo-β-(1→3)-glucanase (E.C. 3.2.1.39)	*Schizosaccharomyces pombe*
CBM 56	Endo-β-(1→3)-glucanase (E.C. 3.2.1.39)	*Bacillus circulans*
		*Bacillus halodurans*
		*Paenibacillus* sp.

**Figure 2:**
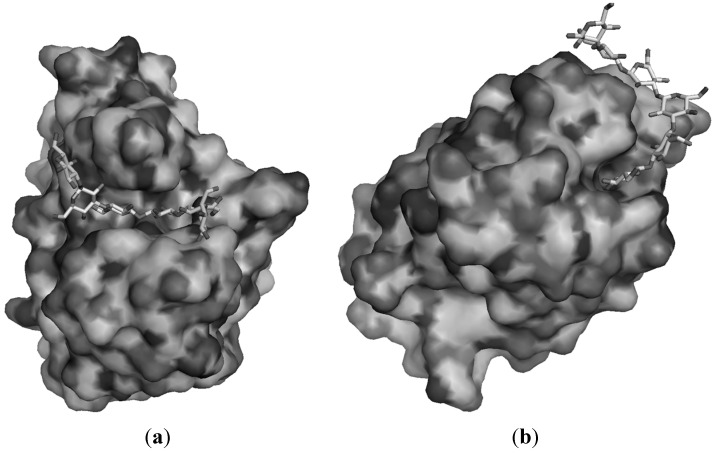
Solvent accessible surface of family 4 CBM from *Thermotoga maritima* complexed with laminarihexaose (**a**) and family 6 CBM from *Bacillus halodurans* complexed with laminarihexaose (**b**) (data from Protein Data Bank entry 1GUI and 1W9W respectively).

CBM family 4 includes proteins with various substrate specificities [xylan, β-(1→4), β-(1→3), β-(1→6) and mixed β-(1→4)-β-(1→3) glucans] [113,114,115]. They are described as lectin like β-jelly roll with shallow cleft on the concave face. Recently, their specificity was studied and it is believed that it arises from the conformation of the cleft. For example, family 4 CBM from *Thermotoga maritima* (*Tm*CBM4) was co-crystallized with laminariheptaose and its three-dimensional structure resolved by molecular replacement with *Cellulomonas fimi* CBM (*Cf*CBM4), a β-(1→4)-glucan receptor (Figure 2) [116]. As *Cf*CBM4, *Tm*CBM4 shows a β-jelly roll structure but differs from the first protein in the geometry of the groove that accommodates the polysaccharide. Indeed, *Cf*CBM4 presents a well-defined linear groove that runs through the protein. Linear β-(1→4)-glucan substrates is sandwiched within an aromatic “cradle” made of three tyrosines and the interaction is further reinforced by a weak network of H-bond with polar amino-acids. A similar organization was identified for the complex laminariheptaose-*Tm*CBM4. The polysaccharide again is trapped between aromatic residues and the aggregate is stabilized by weak H-bond with polar loops found on the sided cliff. Here, the site of interaction adopts a “U-shaped” conformation that perfectly fits the natural conformation of the oligolaminarin. Interestingly, an additional loop, not found in *Cf*CBM4, prevents any linear polysaccharide like β-(1→4)-glucan to fit thus explaining the specificity of the receptors. Therefore, the combination of the unique topology of the groove and the additional loops allow proteins within the same family of CBM to be specific for a particular substrate.

Different conclusions are found with CBM from 6 family specific to β-(1→3)-glucans. In this case, the selectivity for β-(1→3)-glucans is assured thanks to multiple sites of binding, one on the concave face, the other between loops at the edge of the protein. To date, the structure of two family 6 CBM were successfully resolved. The first one is associated with an endo-β-(1→3)-glucanases from *B. halodurans* (*Bh*CBM6, Figure 2) [117]. *Bh*CBM6 binds oligolaminarin with affinity that increases with the length of the substrate. ITC experiment indicates a 1 to 1 ratio between the protein and the sugar and one chain of laminarin probably binds with both sites at the surface of the protein. X-Ray data of the co-crystallized complex laminarihexaose-*Bh*CBM6 was resolved. In contrast to *Tm*CBM4, the oligosaccharide first interacts within a small cleft bounded by a glutamine. This cleft can accommodate only one glucose unit, the non-reducing end that is sandwiched between two tryptophan residues. From there, the oligomer curled around the protein and again adopted a natural “U shape.” At least six binding sub-sites are identified along the trail routed for the sugar and stabilize the whole by direct or water mediated H-bond. Three cations (certainly sodium) reinforce the interaction. As β-(1→4)-glucans adopted in solution a linear conformation, it could not curl like β-(1→3)-glucans but instead it will stick out of the cleft and extend into the solvent. This explains the very low affinity of *Bh*CBM6 for xylans or mixed glucans. The same type of interaction was described for family 6 CBM from *Cellvibrio mixtus*, a saprophytic mesophile soil bacteria [118]. The authors confirmed the stacking of the non-reducing end with hydrophobic residues on one sub-site. The other sub-site was also identified with glutamine as essential residue that contributes mainly through H-bond interaction. Interestingly, this CBM is associated with a cellulase and not a laminarase. Fungi are the main competitors of the *C. mixtus* bacteria for digestion of biomass. *Bh*CBM6 associated with the cellulase might be used to rapidly recruit the glucanase at a site of fungal invasion and thus profit of the lesion to digest easily accessible mixed-glucans or callose. Multiple bindings were also described for family 13 CBM from *Cellulosimicrobium cellulans* where three asparagines and one tryptophan were identified as crucial residues for the interaction [119]. The authors reported cooperative multivalent bindings of laminarin to three different repeat domains on the protein. It allows binding tightly to insoluble long glucans but moderately to shorter oligomers, main products of the glucanases thus avoiding competition. To finish, a CBM from family 43 that bind to β-(1→3)-glucans was recently found associated with a glucan transglycosylase [120]. It shows low similarity with the one found in plants [121]. The binding domain appears to tightly bind to the catalytic domain of the enzyme. The opposite face is thought to be the face of interaction with the glucan.

## 6. Conclusions

This review emphasizes how complex is the elucidation of fine interactions between β-(1→3)-glucans and some known receptors. This is mainly due to the wide variety of glucan structures with really more or less fine structural modulations: chain length, degree and nature of branching, nature of the enchainment of the glucosyl chain (1→3, 1→4, 1→6). This could also be linked with the variety of receptors themselves. This made numerous opportunities to bind, internalize and activate the immune system in order to not only fight opportunistic and pathogenic invasions but also to mimic such an attack by using natural or synthetic glucans as well as synthetic analogues. The latter approach offers a high potential to develop alternative therapeutics. Unfortunately, this review cannot fully focus on the mode of action of small oligo-β-(1→3)-glucans that are however potent immunostimulating agents. It can be hypothesized that analytical methods used to measure interactions with natural receptors are still not sensitive enough, or that other modes of activation of the immune system are indeed involved. As a result, further information may be obtained thanks to the development of new chemical tools, amongst them small glucans and mimetics since they can be synthetically prepared with high degree of purity and high repeatability, criteria difficult to ensure for extracted polysaccharides. Finally, this review also underlined that few crystalline data, generally of high interest to design more efficient drugs, are still required to explain how β-(1→3)-glucans bind mammalian receptors.
